# *miR-1272* Exerts Tumor-Suppressive Functions in Prostate Cancer via *HIP1* Suppression

**DOI:** 10.3390/cells9020435

**Published:** 2020-02-13

**Authors:** Federica Rotundo, Denis Cominetti, Rihan El Bezawy, Stefano Percio, Valentina Doldi, Monica Tortoreto, Valentina Zuco, Riccardo Valdagni, Nadia Zaffaroni, Paolo Gandellini

**Affiliations:** 1 Department of Applied Research and Technological Development, Fondazione IRCCS Istituto Nazionale dei Tumori, 20133 Milan, Italy; federica.rotundo@istitutotumori.mi.it (F.R.); denis.cominetti@unimi.it (D.C.); rihan.elbezawy@istitutotumori.mi.it (R.E.B.); stefano.percio@istitutotumori.mi.it (S.P.); valentina.doldi@istitutotumori.mi.it (V.D.); monica.tortoreto@istitutotumori.mi.it (M.T.); valentina.zuco@istitutotumori.mi.it (V.Z.); 2 Department of Oncology and Hemato-oncology, University of Milan, 20133 Milan, Italy; riccardo.valdagni@istitutotumori.mi.it; 3 Prostate Cancer Program, Fondazione IRCCS Istituto Nazionale dei Tumori, 20133 Milan, Italy; 4 Radiation Oncology 1, Fondazione IRCCS Istituto Nazionale dei Tumori, 20133 Milan, Italy; 5 Department of Biosciences, University of Milan, 20133 Milan, Italy

**Keywords:** prostate cancer, miRNA, HIP1, *miR-1272*, EGFR, radiation, EMT

## Abstract

The development of novel therapies or the improvement of currently used approaches to treat prostate cancer (PCa), the most frequently diagnosed male tumor in developed countries, is an urgent need. In this regard, the functional characterization of microRNAs, molecules shown to regulate a number of cancer-related pathways, is instrumental to their possible clinical exploitation. Here, we demonstrate the tumor-suppressive role of the so far uncharacterized *miR-1272*, which we found to be significantly down-modulated in PCa clinical specimens compared to normal tissues. Through a gain-of-function approach using miRNA mimics, we showed that *miR-1272* supplementation in two PCa cell models (DU145 and 22Rv1) reverted the mesenchymal phenotype by affecting migratory and invasive properties, and reduced cell growth in vitro and in vivo in SCID mice. Additionally, by targeting *HIP1* encoding the endocytic protein HIP1, *miR-1272* balanced EGFR membrane turnover, thus affecting the downstream AKT/ERK pathways, and, ultimately, increasing PCa cell response to ionizing radiation. Overall, our results show that *miR-1272* reconstitution can affect several tumor traits, thus suggesting this approach as a potential novel therapeutic strategy to be pursued for PCa, with the multiple aim of reducing tumor growth, enhancing response to radiotherapy and limiting metastatic dissemination.

## 1. Introduction

Prostate cancer (PCa) represents the most frequently diagnosed cancer in men in the developed countries of Europe and USA [1,2]. Although radical surgery and radiotherapy turned out to be effective to control localized PCa, a significant proportion of patients still experience relapse, in the form of biochemical or local recurrence or metastasis [3]. The efficacy of androgen-deprivation therapy, the treatment of choice for metastatic tumors, is itself limited in time, thus making the management of castration-resistant disease challenging and the patient’s prognosis invariably poor [4]. In this scenario, the development of novel therapies or the implementation of currently used approaches is an urgent need.

MicroRNAs (miRNAs), endogenous small noncoding RNAs that negatively regulate gene expression at the post-transcriptional level [5], have been found aberrantly expressed in several human diseases, including cancer [6,7]. Specifically, depending on their target genes, miRNAs have been acknowledged to exert either pro- or anti-metastatic functions [8].

Based on the available literature, it is still difficult to outline the precise signature of miRNAs related to PCa pathogenesis and progression, however several miRNAs are emerging as master regulators of processes involved in different steps of cancer evolution and in drug response. For example, *miR-29b*, the expression of which is lower in PCa cells compared to normal cells, has been recently reported to inhibit prostate tumor growth in vivo and induce apoptosis by enhancing Bim expression [9]. Down-regulation of *miR-130b* in PCa cells was instead shown to significantly promote the proliferation, invasion and tubule formation of human umbilical vein endothelial cells, while ectopic expression of *miR-130b* blocked prostate cancer angiogenesis in vitro and in vivo [10]. We demonstrated that *miR-205*, a down-regulated miRNA in PCa tissues, is able to pleiotropically hamper PCa metastasis by reverting epithelial to mesenchymal transition (EMT) [11] and restoring the deposition of basement membrane by tumor cells [12]. Consistent with the role of EMT in the establishment of radioresistance [13], *miR-205* can also increase response of PCa cells to ionizing radiation [14]. A tumor-suppressive behavior comparable to that of *miR-205* was reported for *miR-875-5p*, which controls a different set of target genes [15].

Several efforts have been and are still being made to establish miRNA involvement in PCa with the long term perspective to introduce miRNA-based approaches as a novel strategy to withstand the disease, alone or in combination with the currently used therapies. Since the prelude to the development of miRNA-based therapies is the analysis of miRNA function in PCa initiation and progression, in this study, for the first time, we provide evidence of a putative tumor-suppressive role of *miR-1272*.

Specifically, we found that *miR-1272* is down-modulated in PCa samples with respect to normal counterparts. In addition, we showed that, when restored in a couple of metastatic PCa cell lines, *miR-1272* is able to hinder EMT, drastically reduce migration and invasion, limit cell growth and act as radiosensitizer by reducing the levels of Huntingtin Interacting Protein 1 (HIP1), whose overexpression has been associated with PCa and correlated with the severity of the disease.

## 2. Materials and Methods

### 2.1. Cell Culture

Established human PCa cell lines were purchased from American Type Culture Collection (ATCC, Rockville, MD, USA) and cultured in standard conditions. DU145 and 22Rv1 cells were cultured in RPMI-1640 medium (Lonza, Basel, Switzerland) supplemented with 10% FBS (Thermo Fisher Scientific Inc., Waltham, MA, USA). Cell lines were authenticated and periodically monitored by genetic profiling using short tandem repeat analysis (AmpFISTR Identifiler PCR amplification kit, Thermo Fisher Scientific Inc., Waltham, MA, USA). Cells were routinely checked for possible mycoplasma contamination through MycoAlert^®^ Mycoplasma Detection Kit (Lonza, Basel, Switzerland).

Cell morphology was evaluated usually at day 3 after transfection using an Eclipse TE2000-S microscope (Nikon, Japan). Images were acquired by a Digital Camera DXM100F (Nikon, Japan).

### 2.2. Transfection

Cells were seeded at the density of 8000 cells/cm^2^ in culture vessels. Twenty-four hours later, medium was removed and cells were transfected with 20 nM mirVana miRNA mimic (*miR-1272* MC13413, Thermo Fisher Scientific Inc., Waltham, MA, USA) or 30 nM siRNA (*siHIP1*, in sense format 5′-GGCUUAGGAUCGACAAGAA-3′, purchased from Eurofins MWG-Biotech, Ebesberg, Germany) for 4 h, using Optimem medium (Thermo Fisher Scientific Inc., Waltham, MA, USA) and Lipofectamine-2000 reagent (Thermo Fisher Scientific Inc., Waltham, MA, USA) according to the manufacturer’s protocol. A control mirVana miRNA mimic (*miR-Neg* mirVana^TM^ miRNA mimic Negative control #1, Thermo Fisher Scientific Inc., Waltham, MA, USA) and a control siRNA (*siCtr*, in sense format 5′-GCAUACAAUGGAGUUGUUA-3′, purchased from Eurofins MWG-Biotech, Ebesberg, Germany) with no homology to any known human mRNA were used for comparative analyses.

### 2.3. Cell Growth Evaluation

Cells were seeded in 6-well plates at the density of 4000 cells/cm^2^ and transfected with miRNA mimics or siRNAs 24 h later, as previously described. Daily, from day one to five, cells were detached and counted through a Coulter Counter (Beckman Coulter Life Sciences, Brea, CA, USA). Data were reported as cell percentage with respect to the number of *miR-Neg* or *siCtr* cells collected at day 1 (24 h). Cell doubling time of each cell line was calculated from growth curves of parental cells, as described in [16]. Staining for Ki-67 was determined by immunohistochemistry. Briefly, transfected cells were removed from dishes through scraper, formalix-fixed and paraffin-embedded. Some sections were then deparaffinised in xylene, rehydrated through graded alcohols to water, and subjected to immunohistochemical analysis using Ki-67 antibody (MIB-1, Dako; 1:200). Nuclei were counterstained with hematoxylin. Images were acquired by Nikon Eclipse E600 microscope using ACT-1 software (Nikon). At least 10 fields were scanned and the average number of Ki-67-positive and negative cells was plotted.

### 2.4. Apoptosis Analysis

Cell apoptosis was evaluated in terms of catalytic activity of Caspase-3 by using the APOPCYTO Caspase-3 Colorimetric Assay Kit (MBL International Corporation, Woburn, MA, USA), according to manufacturer’s protocol. Briefly, at 96 h after transfection, cells were detached, lysed and extracted proteins were incubated with the substrate N-acetylAsp-Glu-Val-Asp-AMC (DEVD-AMC). The hydrolysis of the proper substrate was evaluated through spectrofluorometry with 380-nm excitation and 460-nm emission filters by using POLARstar OPTIMA plate reader (BMG Labtech, Ortenberg, Germany). For terminal deoxynucleotidyl transferase dUTP nick end labeling (TUNEL) assay, transfected cells were fixed and treated by using the In Situ Cell Death Detection Kit (Roche) according to manufacturer’s instructions. The cells were subjected to FACS analysis (BD Accuri™ C6 Cytometer, Becton Dickinson, Basel CH) and data were reported in graph as the percentage of positive cells.

### 2.5. Migration and Invasion Assays

For migration and invasion assays, cells were cultured and transfected for 72 h as previously described and starved in serum-free medium for 24 h. Cells were transferred to the upper chamber of 24-well Transwell plates (Costar, Corning Incorporated, New York, NY, USA) in serum-free medium at a concentration of 120,000 cells/well. Medium supplemented with 10% of FBS was added to the lower chamber. After a 6 h-incubation at 37 °C, filters were fixed in 99% ethanol and stained with a 0.4% sulforhodamine B/1% acetic acid solution. Migrated cells were counted under a microscope. The same procedure was used for invasion assay, except that cells were seeded at 240,000 cells/well, Transwell chambers coated with 12.5 μg of Matrigel/well (BD Biosciences, San Jose, CA, USA), and samples processed after a 24 h-incubation.

### 2.6. Total RNA Extraction and RT-qPCR

RNA extraction and cDNA synthesis were performed as detailed in [17]. Briefly, RNA was isolated using QIAzol Lysis Reagent and miRNeasy Mini Kit (QIAGEN, Hilden, Germany) with DNase I digestion (QIAGEN, Hilden, Germany) according to the manufacturer’s instructions. cDNA was synthesized using High-Capacity cDNA Reverse Transcription kit (Thermo Fisher Scientific Inc., Waltham, MA, USA). For miRNA detection, TaqMan MicroRNA Reverse Transcription kit and sequence specific primers (Thermo Fisher Scientific Inc., Waltham, MA, USA) were used.

Quantification of gene or miRNA expression was assessed by RT-qPCR as widely described in [16] using No AmpErase TaqMan Universal PCR Master Mix (Thermo Fisher Scientific Inc., Waltham, MA, USA) and the specific TaqMan gene-expression assays (Thermo Fisher Scientific Inc., Waltham, MA, USA), as follows: *miR-1272* (TaqMan^®^ microRNA assay PN4427975), *CDH1* (TaqMan^®^ gene expression assay Hs00170423_m1), *CTNNB1* (TaqMan^®^ gene expression assay Hs00355045_m1), *VIM* (TaqMan^®^ gene expression assay Hs00185584_m1). For comparative analyses, *GAPDH* (PN4326317E) and *RNU48* (TaqMan^®^ microRNA assay PN4440887) were used as endogenous controls for genes and miRNA, respectively. RT-qPCR results were reported as −ddCt or relative quantity (RQ = 2^−ddCt^) with respect to a calibrator sample using the comparative Ct (ddCt) method.

### 2.7. Gene Expression and Bioinformatic Analyses

Transcriptomic profile of DU145 cells transfected with miR-1272 synthetic mimic and those transfected with mock molecule (miR-Neg) were collected in triplicate from scanned images using Illumina BeadStudio v3.3.8 (Illumina) and processed using the lumi package [18] from Bioconductor v3.0 [19]. Raw data were log_2_-transformed, normalized with Robust Spline Normalization and filtered, keeping only probes with a detection *p*-value < 0.01 in at least one sample and probes not associated to official gene symbol were removed. Expression data were deposited in the Gene Expression Omnibus repository (GEO) with accession number GSE68883.

Differentially expressed genes between the two conditions were identified using the *limma* package [20], and significance was assessed by Benjamini–Hochberg false discovery rate (FDR) method in order to take into account the multiple-testing correction. Genes showing an FDR < 0.05 were considered significantly differentially expressed. Selecting only significantly up (fold change, FC > 0) and down regulated (FC < 0) genes an overrepresentation analysis was performed using WebGestalt on-line available tool [21]. Only enrichment with an FDR above 0.05 was considered significant.

miRNA expression data of tumor and non-neoplastic prostate samples from PCa patients were retrieved from GEO repository with accession number GSE76260, in the form of normalized data matrix.

### 2.8. In Silico miR-1272 Target Prediction

Prediction of *miR-1272* target genes was accomplished using miRWalk (http://mirwalk.umm.uni-heidelberg.de/) and MtiBase online tools. The former solely contains in silico predictions from several target-prediction databases while the latter also includes information from experimental data such as ultraviolet cross-linking and immunoprecipitation (CLIP), microarray and RNA sequencing, ribosome-protected fragment sequencing and pulse stable isotope labelling with amino acids in culture (pSILAC) [22]. For miRWalk analysis, target genes were selected as those predicted by at least one database, while for MtiBase at least two prediction databases were considered in association with at least three supporting experiments.

### 2.9. Protein Extraction, Cytoplasmic/Membrane Fractionation and Western Blotting

Total protein extraction and immunoblotting experiments were performed as fully detailed in [16]. In this work the following antibodies were used: AKT (cs9272, Cell Signaling Technologies^®^, Danvers, MA, USA; 1:1000), β-catenin (sc59891, Santa Cruz Biotechnology, Santa Cruz, CA, USA; 1:200), β-tubulin (ab6160, abcam^®^, Cambridge, UK; 1:500), Caveolin-1 (610406, BD Biosciences, San Jose, CA, USA; 1:500), Cleaved PARP (cs9541, Cell Signaling Technologies^®^, Danvers, MA, USA; 1:1000), E-cadherin (sc7870, Santa Cruz Biotechnology, Santa Cruz, CA, USA; 1:500), EGFR (sc03, Santa Cruz Biotechnology, Santa Cruz, CA, USA; 1:200), ERK1/2 (ab17942, abcam^®^, Cambridge, UK; 1:1000), GSK3β (sc9166, Santa Cruz Biotechnology, Santa Cruz, CA, USA; 1:200), HIP1 (MA1-16747, Thermo Fisher Scientific Inc., Waltham, MA, USA; 1:500), PARP (cs9542, Cell Signaling Technologies^®^, Danvers, MA, USA; 1:1000), p-AKT (ab27773, abcam^®^, Cambridge, UK; 1:500), p-ERK1/2 (cs9101, Cell Signaling Technologies^®^, Danvers, MA, USA; 1:1000), p-GSK3β (S9, R&D System, Minneapolis, MN, USA; 1:1000), Slug (cs9585, Cell Signaling Technologies^®^, Danvers, MA, USA; 1:1000), Vimentin (sc32322, Santa Cruz Biotechnology, Santa Cruz, CA, USA; 1:200), Vinculin (V9131, Sigma-Aldrich, St. Louis, MO, USA; 1:5000).

For cytoplasmic/membrane protein separation, the Subcellular Proteins Fractionation Kit for Cultured Cells (Thermo Fisher Scientific Inc., Waltham, MA, USA) was used according to manufacturer’s protocol. Briefly, cultured cells were harvested, treated with buffers to selectively fractionate proteins from cytoplasm (cytoplasmic extraction buffer) and membrane (membrane extraction buffer), and properly centrifuged to enhance protein separation. Immunoblotting experiments were performed as for total proteins.

Quantification of all western blots has been reported in Supplementary Figure S1.

### 2.10. Immunofluorescence

Transfected cells grown for 72 h on μ-Slides 8 Well coverlips (ibidi^®^) were fixed with 4% formaldehyde and permeabilised with cold 70% ethanol. Cells were probed for 1 h with primary antibodies as follows: β-catenin (ab2982, abcam^®^, Cambridge, UK), β-tubulin (ab6046, abcam^®^, Cambridge, UK) and E-cadherin (ab1416, abcam^®^, Cambridge, UK); and subsequently with Alexa Fluor448-labeled or Alexa Fluor594-labeled secondary antibodies (Thermo Fisher Scientific Inc., Waltham, MA, USA) for 1 h at room temperature. Nuclei were counterstained with DAPI (ab104139, abcam^®^, Cambridge, UK; 1:15,000). Images were acquired by Nikon Eclipse E600 microscope using ACT-1 software (Nikon) and processed with ImageJ.

### 2.11. In Vivo Experiments

Experimental protocols were approved by the Ethics Committee for Animal Experimentation of Fondazione IRCCS Istituto Nazionale dei Tumori, and by the Italian Healthy Ministry (project identification code: 1120/2015-PR. Date of approval: 22nd October 2015). Ten million *miR-Neg* or *miR-1272* DU145 cells were subcutaneously injected into the right flank of eight-week-old male SCID mice (Charles River, Calco, Italy). Each group contained ten mice. Animals were maintained under standard light and temperature conditions and had free access to food and water. Tumor growth was determined by measurement of tumor size with a Vernier caliper.

### 2.12. Irradiation Experiments

Irradiation experiments on DU145 cells transfected with *miR-Neg* or *miR-1272* were performed as in El Bezawy et al. [14]. At day 10 after irradiation, colonies were fixed, stained and counted. The colony-forming efficiency was calculated as the ratio of the number of colonies (consisting of at least 50 cells) to the number of single cells seeded. The surviving fraction was calculated as the ratio of the colony-forming efficiency of the irradiated sample to that of the non-irradiated one. Triplicate wells were set up for each condition. Data are reported as a linear-quadratic equation, which has been well established to be a suitable model for the description of cell death upon irradiation [23].

### 2.13. Statistical Analyses

If not otherwise specified, data are presented as mean values +/± SD from at least three independent experiments. Statistical analysis was performed by two-tailed Student’s *t* test except for in vivo experiment where Mann–Whitney U test was applied. *p*-values < 0.05 were considered statistically significant.

## 3. Results

### 3.1. miR-1272 Reverts the Mesenchymal Phenotype and Affects the Migratory and Invasive Properties of PCa Cells

**Figure 1:**
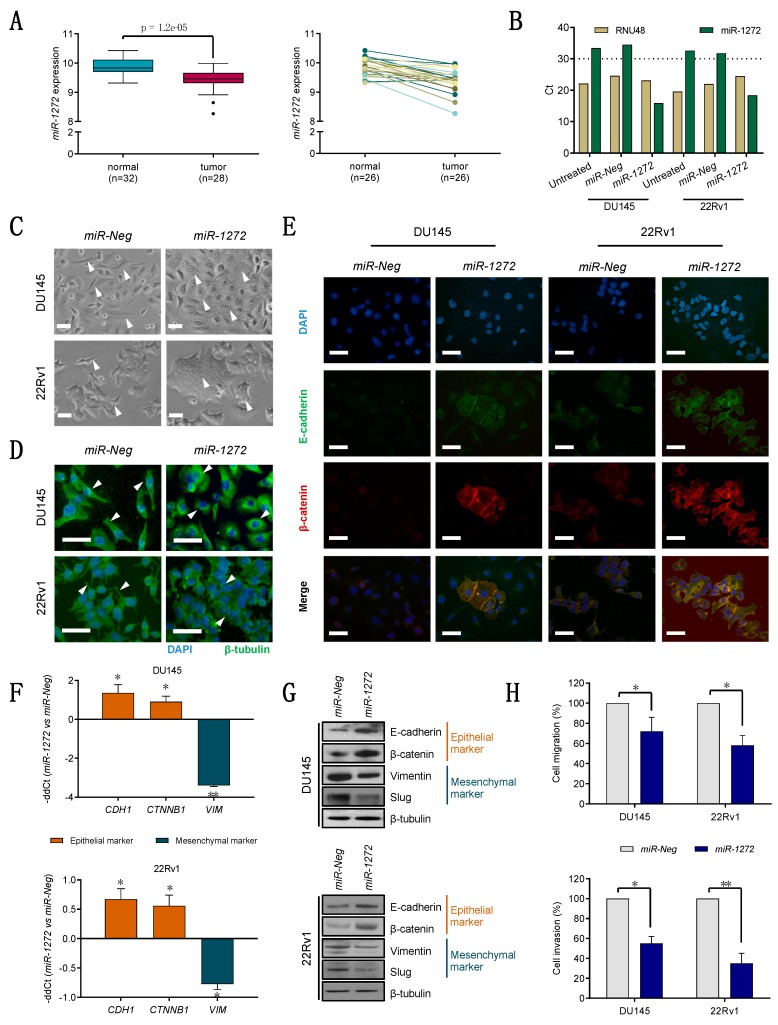
*miR-1272* reverts the mesenchymal phenotype and affects migratory and invasive properties of PCa cells. (**A**) *miR-1272* expression in prostate normal and tumor tissue specimens, as from microarray analysis (left). *miR-1272* expression in matched normal/tumor pairs is reported (right). (**B**) Representative RT-qPCR showing the expression levels of endogenous control *RNU48* (beige columns) and *miR-1272* (green columns) in untreated, *miR-Neg* and *miR-1272* transfected DU145 and 22Rv1 cell lines. Expression values are reported as Cycle threshold (Ct). (**C**) Representative bright-field microphotographs showing morphological changes occurring in *miR-1272* transfected DU145 and 22Rv1 cell lines as compared to *miR-Neg* cells. Scale bar, 50 μm. (**D**) Representative immunofluorescence microphotographs showing the organization of β-tubulin cytoskeleton in *miR-1272* transfected DU145 and 22Rv1 cell lines as compared to *miR-Neg* cells. Scale bar, 50 μm. (**E**) Representative immunofluorescence microphotographs showing E-cadherin (green), β-catenin (red) and their co-localization (yellow merge) at membrane levels in *miR-1272* transfected DU145 and 22Rv1 cell lines as compared to *miR-Neg* cells. Scale bar, 50 μm. (**F**) RT-qPCR showing the relative expression of epithelial (brick red columns) and mesenchymal (petrol blue columns) markers in *miR-1272* transfected DU145 (top) and 22Rv1 (bottom) cells with respect to *miR-Neg* cells, at day 2 after transfection. Expression values are reported as mean −ddCt (+SD) as from 3 independent experiments, using *GAPDH* as endogenous control. (**G**) Immunoblotting showing protein levels of epithelial (brick red) and mesenchymal (petrol blue) markers in *miR-1272* transfected DU145 (top) and 22Rv1 (bottom) cells with respect to *miR-Neg* cells. β-tubulin was used as endogenous control. (**H**) Bar plots reporting the mean percentage (+ SD) of migrated (top) and invaded (bottom) cells in *miR-Neg* (used as control) and *miR-1272* transfected DU145 and 22Rv1 cell lines, as assessed by transwell assay. The results are reported as percentage of *miR-Neg* cells and represent the average of 3 independent experiments. * *p* < 0.05, ** *p* < 0.01, Student′s *t*-test.

By analyzing miRNA expression profiles of prostate clinical samples (GSE76260), we observed that *miR-1272* was significantly down-regulated (FC = −1.34) in 28 tumors compared to 32 normal prostate tissues (both obtained from radical prostatectomies) (Figure 1A, left). In the subset of patients (*n* = 26) with available matched tumor/normal specimens, 85% of cases were characterized by appreciable reduction of *miR-1272* expression levels in cancer tissue compared to the non-neoplastic counterpart (Figure 1A, right).

To investigate the significance of *miR-1272* down-regulation in PCa, we primarily performed in vitro gain-of-function experiments in two PCa cell lines (DU145 and 22Rv1) that express only barely detectable *miR-1272* (Ct > 30) (Figure 1B), consistent with the data obtained on patients’ specimens. When orthotopically injected, DU145 cells have the ability to form metstatic nodes in the peritoneal surface and diaphragm [24]. Expression of *miR-1272* was efficaciously restored in both cell lines using a synthetic mimic (*miR-1272*), as compared to cells transfected with a negative molecule (*miR-Neg*) (Figure 1B).

The most evident phenotypic alteration upon *miR-1272* supplementation was a marked morphological change, consisting of an increase of cell size together with loss of typically mesenchymal morphological features and the acquisition of a more epithelial aspect (Figure 1C). Specifically, *miR-1272* DU145 cells showed a decrease in the fraction of spindle-like cells and a parallel increase in that of enlarged, polygonal shaped and flattened cells with poorly defined profiles (Figure 1C, top). In *miR-1272* 22Rv1 cells, we observed the formation of syncytium-like structures, which made cell profiles almost indiscernible, indicating that cells tended to aggregate in tightly packed structures probably due to enhanced cell-cell adhesion (Figure 1C, bottom). Moreover, β-tubulin staining highlighted cytoskeletal redistribution: in *miR-Neg* DU145 cells, microtubules were oriented parallelly to the cortex, whereas they displayed a radial pattern of organization in *miR-1272* DU145 cells (Figure 1D, top); in the 22Rv1 model, the filopodia and lamellipodia staining of *miR-Neg* cells was lost in the tightly embedded *miR-1272* cells (Figure 1D, bottom). In addition, E-cadherin and β-catenin (which stabilizes E-cadherin complex) showed a strong co-localization at the cell membrane in *miR-1272* compared to *miR-Neg* cells (Figure 1E), in both PCa models. Altogether, these changes comply with the evidence that *miR-1272* restoration can induce a transition from a mesenchymal-like toward a more epithelial-like phenotype, as supported by the over-expression of the epithelial markers (E-cadherin and β-catenin) and the down-modulation of the mesenchymal ones (vimentin and slug) at mRNA and/or protein levels (Figure 1F,G). Coherent with the reversion of the mesenchymal phenotype, *miR-1272* cells showed significantly impaired migratory (Figure 1H, top) and invasive (Figure 1H, bottom) capabilities compared to *miR-Neg* cells, suggesting the involvement of *miR-1272* loss in the initial phases of the metastatic process, specifically EMT, migration and invasion.

### 3.2. miR-1272 Reduces the Growth of PCa Cell Lines Both In Vitro and In Vivo

**Figure 2:**
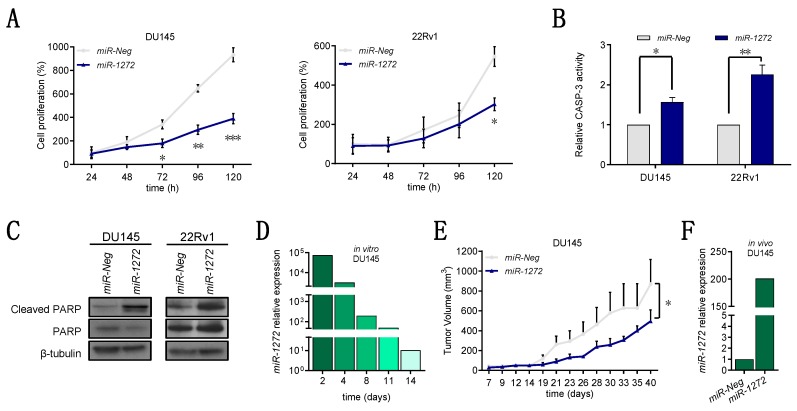
*miR-1272* reduces the growth of PCa cell lines both in vitro and in vivo. (**A**) Graphs reporting the growth of *miR-Neg* and *miR-1272* transfected DU145 (top) and 22Rv1 (bottom) cells at different time points after transfection. Data are reported as percentage (mean ± SD as from 3 independent experiments) of cell number in each condition with respect to the number of *miR-Neg* cells collected at 24 h. (**B**) Bar plots reporting the relative catalytic activity of Caspase-3 (mean + SD from 3 independent experiments), an indicator of apoptosis induction, at 96 h after transfection of DU145 and 22Rv1 cells with *miR-Neg* and *miR-1272*. (**C**) Representative immunoblotting showing protein levels of cleaved and total PARP in *miR-Neg* and *miR-1272* transfected DU145 (left) and 22Rv1 (right) cells. β-tubulin was used as endogenous control. (**D**) RT-qPCR showing relative expression of *miR-1272* in *miR-1272* transfected DU145 cells with respect to *miR-Neg* cells, at different time points after transfection. *RNU48* was used as endogenous control. (**E**) Graph reporting the tumor growth volume (mean + SD) of xenografts originated from *miR-Neg* and *miR-1272* transfected DU145 cells (10 mice per group), as measured with a Vernier caliper on the indicated days after cell injection into SCID mice. (**F**) RT-qPCR showing relative expression of *miR-1272* in xenografts derived from *miR-1272* transfected DU145 cells as compared to those derived from *miR-Neg* cells, at 23 days after injection into SCID mice. *RNU48* was used as endogenous control. * *p* < 0.05, ** *p* < 0.01, *** *p* < 0.001, Student’s *t*-test.

Aside from *miR-1272* capability to revert EMT, we noticed that *miR-1272*-transfected cells were markedly reduced in number when compared to the *miR-Neg*-transfected counterpart. Through daily evaluation from one day after the transfection until five days, we found that *miR-1272* cell number increased more slowly than that of *miR-Neg* cells in both PCa models, though statistically significant differences could be appreciated in 22Rv1 cells only at the end of the experiment (Figure 2A). Consistent with this, *miR-1272* reduced the number of Ki-67-positive cells in both cell lines, although at a different extent, which is indicative of a reduced proportion of actively proliferating cells (Supplementary Figure S2A). We asked whether such cell number reduction could be attributed to apoptosis induction. In this regard, we found a significant increase in the catalytic activity of Caspase-3 in both *miR-1272* over-expressing PCa models at 96 h after transfection (Figure 2B), as well as augmented cleavage of PARP, which commands the down-stream activation of the apoptotic process (Figure 2C). Consistent with this, TUNEL assay showed increased number of apoptotic cells in *miR-1272* transfectants in both cell lines (Supplementary Figure S2B). We reasoned that the latency between apoptosis induction (four days) and evidence of significantly reduced cell number (five days) in 22Rv1 cells may depend on the longer doubling time of these cells as compared to DU145 cells (doubling time of 39.39 vs. 29.96).

Moreover, to elucidate the behavior of *miR-1272*-reconstituted cells in the in vivo context, we subcutaneously injected *miR-1272* or *miR-Neg* DU145 cells in the flank of male SCID mice. To ensure that injected cells could express *miR-1272* for sufficient time to impact on xenograft take and growth, we had preliminarily verified in vitro that *miR-1272* DU145 cells maintained significantly higher miRNA levels than *miR-Neg* cells for at least 14 days (Figure 2D). In line with in vitro cell growth data, *miR-1272* supplementation resulted in a slower tumor growth in vivo, though not affecting xenograft take. At day 23 after cell injection, *miR-1272* was still about 200-fold more expressed in *miR-1272* xenografts as compared to *miR-Neg* ones (Figure 2F). As a whole, these results provide evidence about the putative involvement of *miR-1272* in the impairment of PCa cell growth, which at least in part relies on the induction of apoptotic cell death.

### 3.3. HIP1 Is a Functional Target of miR-1272

**Figure 3:**
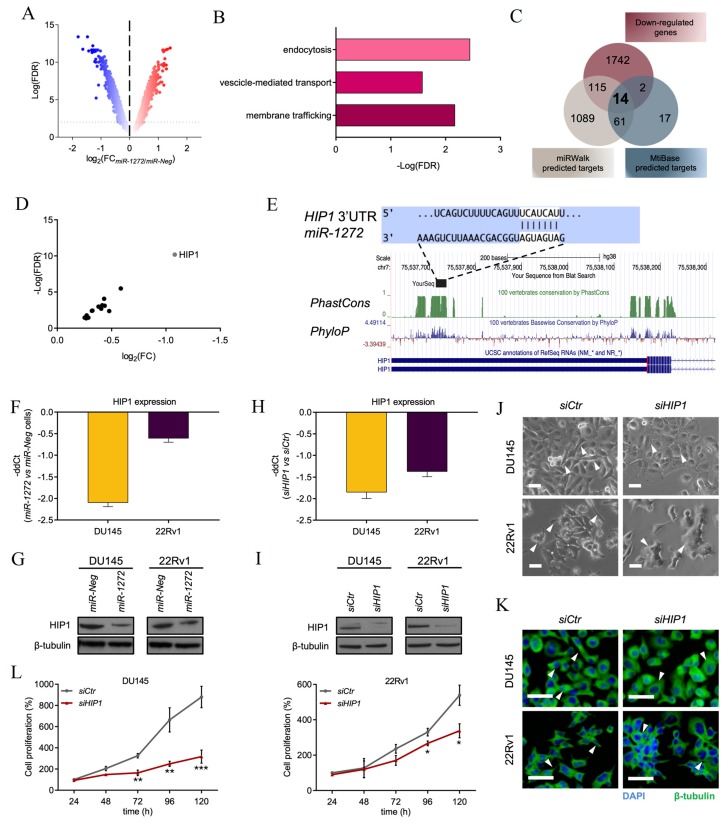
*HIP1* is a functional target of *miR-1272.* (**A**) Volcano plot showing fold-change (FC) and false discovery rate (FDR) of differentially expressed genes (red: up, blue: down) in *miR-1272* transfected DU145 cells as compared to *miR-Neg* cells, as measured by microarray analysis. (**B**) Graph reporting FDR values of pathways enriched in gene ontology analysis carried out on genes differentially expressed genes upon *miR-1272* supplementation. (**C**) Venn diagram indicating the number of genes significantly down-regulated in microarray analysis of *miR-1272* cells and the number of genes predicted to be targets of *miR-1272* as from miRWalk or MtiBase. The fourteen genes shared among the 3 lists are included in Table 1. (**D**) Plot reporting FC and FDR values of the 14 genes emerging from intersections.(**E**) Representation of *miR-1272* duplexed with the 3′UTR of *HIP1* mRNA, as from TargenScanHuman (http://www.targetscan.org/vert_72/) (top). Conservation of the miRNA binding site across vertebrates is made evident by graphs of PhastCons and PhyloP scores, as from UCSC Genome Browser (https://genome.ucsc.edu) (bottom). (**F**) RT-qPCR showing the relative expression of *HIP1* in *miR-1272* transfected DU145 and 22Rv1 cells with respect to *miR-Neg* cells, at day 2 after transfection. Expression values are reported as −ddCt (+SD) as from 3 independent experiments, using *GAPDH* as endogenous control. (**G**) Representative immunoblotting showing protein levels of HIP1 in *miR-Neg* and *miR-1272* transfected DU145 (left) and 22Rv1 (right) cells. β-tubulin was used as endogenous control. (**H**) RT-qPCR showing the relative expression of *HIP1* in *siHIP1*-transfected DU145 and 22Rv1 cells with respect to *siCtr* cells, at day 2 after transfection. Expression values are reported as −ddCt (+ SD) as from 3 independent experiments, using *GAPDH* as endogenous control. (**I**) Representative immunoblotting showing protein levels of HIP1 in *siCtr* and *siHIP1* transfected DU145 (left) and 22Rv1 (right) cells. β-tubulin was used as endogenous control. (**J**) Representative bright-field microphotographs showing morphological changes occurring in *siHIP1* transfected DU145 and 22Rv1 cell lines as compared to *siCtr* cells. Scale bar, 50 μm. (**K**) Representative immunofluorescence microphotographs showing the organization of β-tubulin cytoskeleton in *siHIP1* transfected DU145 and 22Rv1 cell lines as compared to *siCtr* cells. Scale bar, 50 μm. (**L**) Graphs reporting the growth of *siCtr* and *siHIP1* transfected DU145 (left) and 22Rv1 (right) cells at different time points after transfection. Data are reported as percentage (mean ± SD as from 3 independent experiments) of cell number in each condition with respect to the number of *siCtr* cells collected at 24 h. * *p* < 0.05, ** *p* < 0.01, *** *p* < 0.001, Student’s *t*-test.

To start understanding the mechanisms underlying the tumor-suppressive role of *miR-1272* in PCa, gene expression profiles of DU145 cells transfected with *miR-1272* mimic were comparatively evaluated to those of *miR-Neg* cells on a microarray platform. Differential expression analysis (FDR < 0.05) revealed 1804 and 1873 genes respectively up- and down-regulated in *miR-1272* cells (Figure 3A). Over-representation analysis performed on such genes revealed a significant enrichment in “membrane trafficking” (FDR = 6.71 × 10^−3^) and “vescicle-mediated transport” (FDR = 2.63 × 10^−2^) annotations of REACTOME and in “endocytosis” (FDR = 3.57 × 10^−3^) annotations of KEGG pathway collections (Figure 3B).

In search for potential *miR-1272* direct targets, down-regulated genes were crossed with the lists of predicted targets from miRWalk and MtiBase tools. The latter associates in silico predictions (obtained from five different miRNA target prediction tools) to experimental data from CLIP-Seq and technical validations. Overall, the resulting intersection included 14 candidate genes (Figure 3C), which are listed in Table 1 and plotted in Figure 3D.

The most significantly down-modulated target gene with the highest number of experimental validations resulted to be *HIP1*. One site complementary to *miR-1272* seed is actually evident in position from 450 to 456 of *HIP1* 3′ UTR (Figure 3E, top). Notably, such sequence revealed to be conserved through vertebrates with PhyloP and PhastCons scores comparable to those of *HIP1* last coding exon (Figure 3E, bottom). Intriguingly, HIP1 has been found significantly over-expressed in PCa [25] and reported to be involved in the down-modulation of several membrane receptors associated with a more aggressive behavior in a broad spectrum of human epithelial cancers [26]. Functionally, HIP1 regulates membrane trafficking by controlling the correct turn-over of membrane receptors and their resulting endocytosis and/or degradation [27]. Altogether these aspects prompted us to validate *HIP1* as a potential *miR-1272* target. In this regard, we confirmed repression of HIP1 at both mRNA (Figure 3F) and protein (Figure 3G) level in both *miR-1272*-transfected DU145 (consistent with gene expression data) and 22Rv1 cell lines.

To corroborate our hypothesis, we adopted a phenocopy strategy by using a specific siRNA against *HIP1* (hereafter *siHIP1*). Primarily, we tested the efficacy of the *siHIP1*, which revealed to markedly down-modulate HIP1 at mRNA (Figure 3H) and, consequently, at protein level (Figure 3I). RNAi-mediated *HIP1* knock-down was able to recapitulate several aspects of *miR-1272* reconstituted phenotype. Specifically, morphological changes occurring in PCa cells depleted for the expression of *HIP1* paralleled those of cells restored for *miR-1272*, in terms of appearance of more enlarged and tightly embedded cells (Figure 3J) as well as cytoskeletal re-organization (Figure 3K). *HIP1*-silenced cells also showed a significantly decreased cell growth (Figure 3L), which was appreciable starting from 72 h upon siRNA transfection in both PCa models, although to a different extent, in line with the effect produced by *miR-1272* supplementation.

Overall, this evidence supports the hypothesis that at least part of *miR-1272* tumor-suppressive effects are maybe ascribable to its ability to directly repress *HIP1*.

### 3.4. miR-1272 Affects EGFR/AKT/ERK Signaling and Increases Ionizing Radiation Response

**Figure 4:**
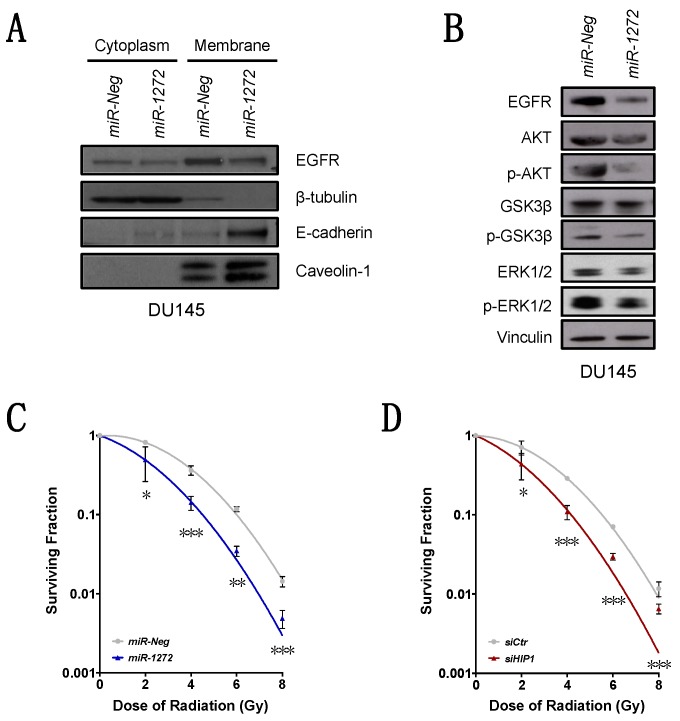
*miR-1272* affects EGFR/AKT/ERK signaling and increases ionizing radiation response. (**A**) Representative immunoblotting showing protein levels of cytoplasmic and membrane-associated EGFR and E-cadherin in *miR-Neg* and *miR-1272* transfected DU145 cells. β-tubulin and Caveolin-1 were used as controls for cytoplasm/membrane fractionation. (**B**) Representative immunoblotting showing protein levels of EGFR/AKT/GSK3β/ERK pathway members in *miR-Neg* and *miR-1272* transfected DU145 cells. Vinculin was used as endogenous control. (**C**) Clonogenic cell survival of DU145 cells transfected with *miR-Neg* or *miR-1272* and exposed to increasing doses of irradiation (2, 4, 6 and 8 Gy). The surviving fraction is reported as mean ± SD values from 3 independent experiments. (**D**) Clonogenic cell survival of DU145 cells transfected with *siCtr* or *siHIP1* and exposed to increasing doses of irradiation (2, 4, 6 and 8 Gy). The surviving fraction is reported as mean ± SD values from 3 independent experiments. * *p* < 0.05, ** *p* < 0.01, *** *p* < 0.001, Student’s *t*-test.

HIP1 over-expression is often associated with a general unbalanced membrane receptor trafficking [26]. This aberration has been shown to promote tumor formation and lead to the amplification of the downstream signaling of growth factor receptors, among which is Epidermal Growth Factor Receptor (EGFR) [26]. In this regard, we evaluated EGFR membrane levels upon *miR-1272* supplementation and observed that they were reduced in both *miR-1272* PCa models (Figure 4A, Supplementary Figure S3A). Total EGFR amount was significantly down-modulated at protein level in both *miR-1272* transfected PCa cell models (Figure 4B), suggesting a putative involvement of HIP1 in regulating EGFR protein turnover. Additionally, the EGFR/AKT/ERK axis (known to be involved in the regulation of cell growth, EMT and metastasis [27]) was found to be affected in *miR-1272*-restored cells (Figure 4B, Supplementary Figures S1H and S3B). Specifically, in DU145 cells, a feeble reduction of AKT expression was accompanied by a marked down-modulation of its active form, p-AKT, which in turn, could not act on its targets. Consistently, in *miR-1272* cells we found lower levels of p-GSK3β, which is the inactive form of the pro-apoptotic protein GSK3β (Figure 4B). In parallel, ERK1/2 were found to be markedly reduced in their active phosphorylated forms in both DU145 and 22Rv1 cells, though in the latter model no major alterations of other components of AKT/GSK3β axis were observed (Figure 4B, Supplementary Figures S1H and S3B).

Given that radiotherapy represents one of the main treatment choices for organ-confined PCa, and based on the emerging evidence indicating miRNA involvement in EGFR-mediated radiosensitization in PCa models [14,15], we investigated whether *miR-1272* supplementation could modify the sensitivity of DU145 cells to ionizing radiation. We found that *miR-1272* was actually able to radiosensitize PCa cells, as suggested by the consistent reduction of clonogenic cell survival of *miR-1272*-reconstituted DU145 cells upon irradiation, as compared to *miR-Neg* cells (Figure 4C). Consistent with the evidence that *HIP1* is a functional target of *miR-1272*, *HIP1* silencing by siRNA phenocopied the increased radiosensitivity of *miR-1272*-transfected cells (Figure 4D).

Altogether these results let us to speculate about the tumor-suppressive role of *miR-1272* in PCa context, by acting (albeit in a cell-model-dependent manner) mainly on EGFR turnover/membrane exposition, and subsequently on EGFR downstream pathways controlling cell growth, apoptosis, EMT and radiation response.

## 4. Discussion

Compelling evidence about the involvement of endocytic proteins in cancer initiation and progression has emerged recently [26]. However, the exact mechanism by which a disrupted regulation of the endocytic pathway may lead a normal cell to become cancerous is still completely unclear [26]. Several proteins are involved in endocytosis/clathrin-mediated pathways and in turn copious membrane-protein (e.g., growth factor receptors) turn-over is finely regulated by these mechanisms. In this regard, an impaired/unbalanced receptor-trafficking mediated by endocytosis has been associated to cell transformation and cancer [26].

Chiefly, HIP1, a cofactor in clathrin-mediated vescicle trafficking, was firstly found to be implicated in cancer as part of a chromosomal translocation in patients with chronic myelomonocytic leukemia [28]. Other studies revealed HIP1 to be deregulated in several human solid tumors [26]. Particularly, HIP1 expression was demonstrated to be more elevated in prostate and colon cancers with respect to their normal counterparts. In addition, HIP1 expression increased in parallel with PCa progression up to metastasis. Consistent with this evidence, primary prostate tumors lacking HIP1 expression were characterized by reduced frequency of disease progression, in terms of biochemical relapse, after radical prostatectomy [26]. Altogether, these findings suggested HIP1 as a putative prognostic biomarker and therapeutic target for PCa management [26].

Different layers of regulation may impact on HIP1 expression. In the last decades, miRNAs have gained increasing respect as regulators of various biological processes [29]. Hence, aberrant miRNA expression has been causatively associated with the pathogenesis of several diseases, including cancer [7]. Depending on the cellular context and the functions of their targets, miRNAs may act as oncomirs or tumor-suppressors. Generally, the down-modulation of a tumor-suppressor miRNA capable of controlling the expression of an oncogene may result in deregulated over-expression of the target, thus contributing to tumor development and progression [30]. Several tumor-suppressive miRNAs have been identified in PCa context, and their role clarified through functional studies. However, the function of *miR-1272* in PCa, and in human cancer in general, has not been explored so far. Here, we show that *miR-1272* is downregulated in PCa compared to adjacent normal tissue. The reconstitution of *miR-1272* expression in PCa cells, inherently expressing negligible levels of the miRNA, resulted in a marked abrogation of its predicted target *HIP1*. Notably, the results of 6 Ago-CLIP sequencing experiments collected in Mtibase (Table 1) confirmed that *miR-1272* binding site within *HIP1* 3′UTR is not merely predicted in silico but is also physically bound by Ago proteins within RNA-induced silencing complex. Such evidence confirms the functional repression of *HIP1* by *miR-1272*, though not relying on the conventional luciferase reporter assays. Our results pave the way for future studies aimed at verifying whether pathological loss of *miR-1272* in normal cells may lead to HIP1 over-expression, which in turn may dysregulate the endocytic pathway, ultimately disrupting signalling pathways that control growth, survival and migration.

Aberrant endocytic turnover may lead to the amplification of signal transduction cascades mediated by a sustained activation of growth factor receptors (e.g., EGFR). The upregulation of EGFR signalling has been widely associated with more aggressive behaviour in several human cancers [31]. In addition, in the context of PCa, the acquisition of a hormone-refractory state was suggested to be related to an excessive activation of the EGFR axis and the downstream pathways, including PI3K-AKT and ERK, thus impinging on several tumor traits [32]. In our PCa models, *miR-1272* supplementation significantly reduced EGFR membrane exposition, presumably as a consequence of the restored correct receptor turn-over. Interestingly, such phenotype was recapitulated by the direct silencing of *HIP1*. *MiR-1272*-mediated impairment of EGFR signalling resulted in reduced activation of ERK1/2 and AKT-GSK3β axes, which ultimately led to reduced cell growth and migration, respectively. Similar results were reported in the context of fibroblast-like synoviocyte models of rheumatoid arthritis, one of the most common autoimmune diseases, where HIP1 expression was shown to be required for receptor tyrosine kinase (RTK) stabilization on the cell surface. In this regard, HIP1 abrogation was able to reduce fibroblast-like synoviocyte invasion proficiency by interfering with lamellipodia formation and cytoskeletal re-organization, a phenotype sustained by the deregulation of ERK1/2 signalling upon HIP1 silencing [33]. Again, during angiogenesis (*i.e*., the formation of new blood vessels from the pre-existing ones) for tissue repair in various pathological conditions such as ischemic cardiovascular events, endothelial cells are stimulated by VEGF to proliferate and migrate. In this context, *miR-135a-3p* was shown to be able to impair p38 signalling in response to VEGF stimulation through the suppression of HIP1. In addition, endothelial cell growth and migration resulted severely affected upon *miR-135a-3p* restoration [34]. Hence, the disruption of membrane receptor turn-over mediated by HIP1 shows comparable phenotypes and involves similar pathways in completely different settings. In the context of Huntington Disease (HD), another epigenetic regulator of *HIP1* and of other HD-related factors is *miR-128a* [35]. The same miRNA was identified upon an integrative analysis of proteomic changes during PCa progression. The proteomic signature revealed the involvement of *miR-128a* (and *miR-128b*) in regulating the transition from organ-confined disease to metastasis. Precisely, *miR-128* levels were reduced in invasive PCa compared to benign prostate epithelial, and *miR-128* overexpression was able to attenuate invasion of PCa cells [36]. Comprehensively, miRNAs capable (or supposed) to target *HIP1* were demonstrated to impair/affect migration and invasion, the prelude of which (in epithelial tumors) is EMT.

In this study, we elucidated the role of *miR-1272* as repressor of EMT process. Aside from being a crucial step for the metastatic dissemination, which represents the main cause of death in PCa patients, EMT was reported as related to radioresistance in several tumors [37]. In PCa context, radiation therapy represents a standard of care whereas radiation resistance remains the main obstacle for long-term treatment efficacy. In fact, upon radiation, cancer cells activate several different pathways to overcome cytotoxic effects of treatment. In breast cancer, ionizing radiation was found to enable HER RTKs and the downstream pathways such as AKT and ERK1/2 [38]. The pro-survival effects mediated by the aforementioned pathways are related to the abolishment of the apoptotic stimuli and, concurrently, to the induction of cell cycle checkpoint response and DNA repair. In this regard, similar mechanisms of miRNA-mediated radiosensitization through RTKs have been recently described in PCa models. Primarily, *miR-205*, the role of which elicits great interest in PCa, was demonstrated to be involved in regulating EGFR nuclear-translocation, a mechanism known to control ionizing radiation response, by targeting PKCε [14]. In addition, in both prostate and breast cancers, *miR-205* revealed to be capable to enhance radiation response by targeting ZEB1, a well-established EMT inhibitor [14,15,16,17,18,19,20,21,22,23,24,25,26,27,28,29,30,31,32,33,34,35,36,37,38,39]. Similarly, another miRNA, *miR-875-5p*, was found to radiosensitize PCa cells by targeting EGFR and thereby reducing receptor nuclear-translocation [15]. Altogether, these findings suggest that the complex network existing between miRNAs, EGFR, ERK1/2 axis and EMT process is involved in radioresistance. Here, we proved, for the first time, the radiosensitizing effect of *miR-1272* in PCa, and showed that this effect is at least in part mediated by *HIP1* suppression.

Overall, a putative therapeutic approach based on *miR-1272* reconstitution in association with ionizing radiation may improve PCa response to conventional radiotherapy, as we showed that *miR-1272* can reduce the activity of EGFR/AKT/ERK1-2 pathways, known to be triggered as pro-survival signals upon irradiation. A potential clinical interest for *miR-1272* may also lie on its ability to affect cell growth and EMT. In this regard, a *miR-1272*-based therapy in an organ-confined PCa context may primarily represent a suitable strategy to reduce tumor growth and enhance response to radiotherapy. Additionally, *miR-1272* may also prevent EMT, the first step of metastatic cascade. Prerequisite to the development of any *miR-1272*-based therapeutic approach would be the setting up of miRNA mimic delivery strategies streamlined for efficiency and specificity of prostate targeting.

## Figures and Tables

**Table 1:** Fourteen bona fide *miR-1272* targets.

Symbol	CLIP-seq *	Gene Name	logFC	FDR
*HIP1*	6	huntingtin interacting protein 1	−1.08	6.66 × 10^−11^
*BCL11A*	5	B-cell CLL/lymphoma 11A (zinc finger protein)	−0.53	7.79 × 10^−5^
*FOXK2*	8	forkhead box K2	−0.48	4.22 × 10^−3^
*ATF6*	3	activating transcription factor 6	−0.44	7.87 × 10^−4^
*SMARCA2*	4	SWI/SNF related, matrix associated, actin dependent regulator of chromatin, subfamily a, member 2	−0.43	8.57 × 10^−5^
*CERS6*	2	ceramide synthase 6	−0.41	6.79 × 10^−4^
*ZNRD1*	3	zinc ribbon domain containing 1	−0.40	2.01 × 10^−3^
*MRPL9*	10	mitochondrial ribosomal protein L9	−0.38	1.03 × 10^−3^
*GHITM*	4	growth hormone inducible transmembrane protein	−0.32	3.74 × 10^−3^
*DAPK1*	2	death-associated protein kinase 1	−0.32	3.89 × 10^−3^
*PURB*	7	purine-rich element binding protein B	−0.29	3.51 × 10^−2^
*TTC5*	2	tetratricopeptide repeat domain 5	−0.27	3.84 × 10^−2^
*BCL2L2*	3	BCL2-like 2	−0.27	1.72 × 10^−2^
*HTRA1*	2	HtrA serine peptidase 1	−0.25	3.47 × 10^−2^

* number of CLIP-seq experiments supporting the association between Ago proteins and *miR-1272* binding site within the 3′UTR of the indicated gene.

## Supplementary Materials

The following are available online at https://www.mdpi.com/2073-4409/9/2/435/s1, Figure S1: The figure reports quantification of all western blots. Densitometry data are reported as raw normalized values (arbitrary units) towards the loading control of each blot. Figure S2: Plots indicating the percentages of (A) Ki-67-positive and negative cells and (B) TUNEL-positive cells in *miR-Neg* and *miR-1272*-transfected DU145 and 22Rv1 cells, Figure S3: (A) Representative immunoblotting showing protein levels of cytoplasmic and membrane-associated EGFR in *miR-Neg* and *miR-1272*-transfected 22Rv1 cells. β-tubulin and E-cadherin were used as controls for cytoplasm/membrane fractionation. (B) Representative immunoblotting showing protein levels of EGFR/AKT/GSK3β/ERK pathway members in *miR-Neg* and *miR-1272*-transfected 22Rv1 cells. Vinculin was used as endogenous control.
