# Supplementary material for: miR-1272 Exerts Tumor-Suppressive Functions in Prostate Cancer via HIP1 Suppression
Source: Cells. 2020 Feb 13;9(2):435. doi: 10.3390/cells9020435 (PMC7072756; doi:10.3390/cells9020435)
Supplement: Supplementary file 1 [file cells-09-00435-s001.pdf]

## Supplementary Figures

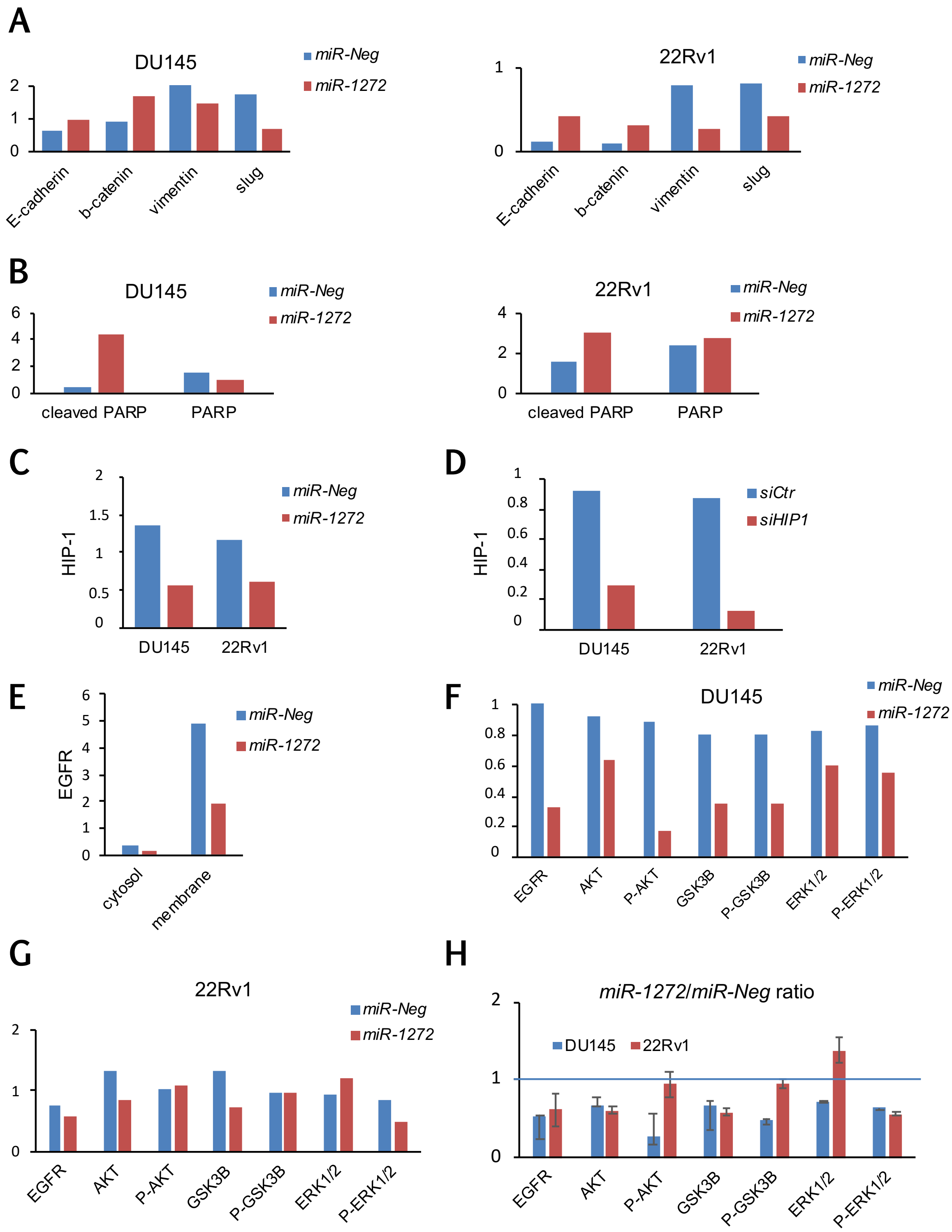


**Supplementary Figure 1**
The figure reports quantification of all western blots. Densitometry data are reported as raw normalized values (arbitrary units) towards the loading control of each blot. (**A**) related to Fig. 1G. Loading control: β-tubulin; (**B**) related to Fig. 2C. Loading control: β-tubulin; (**C**) related to Fig. 3C. Loading control: β-tubulin; (**D**) related to Fig. 3I. Loading control: β-tubulin; (**E**) related to Fig. 4A. Loading control: β-tubulin for cytoplasm and caveolin-1 for the membrane; (**F**) related to Fig. 4B. Loading control: vinculin; (**G**) related to Supplementary Fig. 3B. Loading control: vinculin; (**H**) *miR-1272/miR-Neg* ratios for proteins shown in Fig. 4B and Supplementary Fig. 3B, reported as mean + sd from 3 independent western blots.

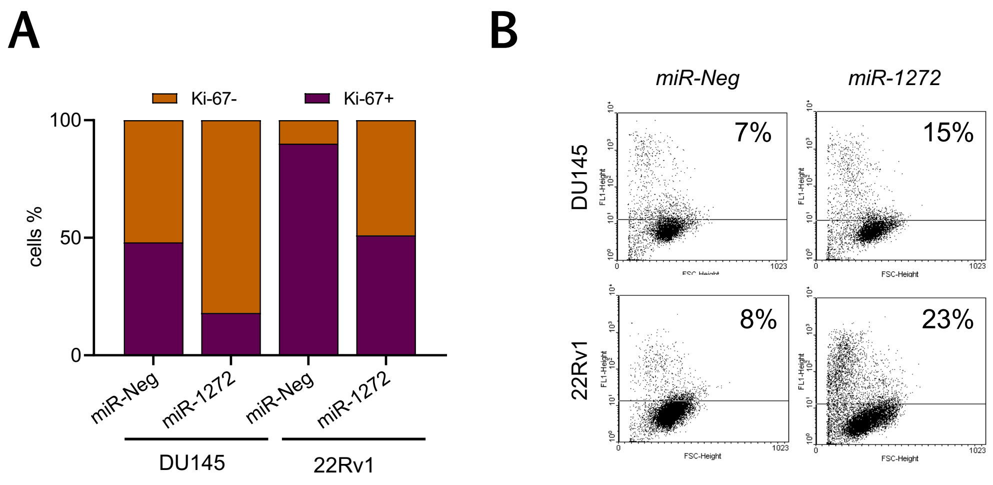


**Supplementary Figure 2**

Plots indicating the percentages of (**A**) Ki-67-positive and negative cells and (**B**) TUNEL-positive cells in *miR-Neg* and *miR-1272*-transfected DU145 and 22Rv1 cells.

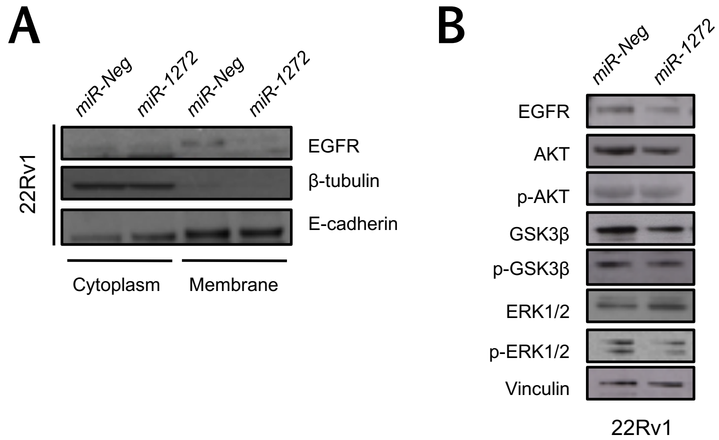


**Supplementary Figure 3**
(**A**) Representative immunoblotting showing protein levels of cytoplasmic and membrane-associated EGFR in *miR-Neg* and *miR-1272*-transfected 22Rv1 cells. β-tubulin and E-cadherin were used as controls for cytoplasm/membrane fractionation. (**B**) Representative immunoblotting showing protein levels of EGFR/AKT/GSK3β/ERK pathway members in *miR-Neg* and *miR-1272*-transfected 22Rv1 cells. Vinculin was used as endogenous control.
